# Discovery of Novel and Clinically Relevant Markers in Formalin-Fixed Paraffin-Embedded Esophageal Cancer Specimen

**DOI:** 10.3389/fonc.2018.00157

**Published:** 2018-05-09

**Authors:** Joe Abdo, Christopher S. Wichman, Nicholas E. Dietz, Pawel Ciborowski, John Fleegel, Sumeet K. Mittal, Devendra K. Agrawal

**Affiliations:** ^1^Department of Clinical and Translational Science, Creighton University School of Medicine, Omaha, NE, United States; ^2^Department of Biostatistics, College of Public Health, University of Nebraska Medical Center, Omaha, NE, United States; ^3^Department of Pathology, CHI Health Creighton University Medical Center, College of Medicine, Omaha, NE, United States; ^4^Department of Pharmacology, University of Nebraska Medical Center, Omaha, NE, United States; ^5^Norton Thoracic Institute, St. Joseph’s Hospital and Medical Center, Dignity Health, Phoenix, AZ, United States

**Keywords:** mass spectrometry, formalin-fixed paraffin-embedded tissue, SWATH analysis, proliferation markers, proteomics, molecular oncology, chemoresistance markers

## Abstract

Due to the ineffectiveness of chemoradiation and targeted therapy in esophageal anticancer care and the subsequent low survival rates, we constructed a high throughput method to discover and investigate new markers with prognostic, diagnostic, and therapeutic clinical utility. This was accomplished by developing a quick, inexpensive, and dependable platform to simultaneously quantify thousands of proteins which subsequently revealed novel markers involved in the pathogenesis of esophageal adenocarcinoma (EAC) *via* discovery mass spectrometry paired with conservative biostatistics. Our method uncovered a perfect storm of tumor suppressors being downregulated, proliferation markers ramped up, and chemoresistance markers overexpressed—many of which could serve as new therapy targets for EAC. The 12 markers discovered by this method are novel regarding their involvement in the pathogenesis of EAC. The molecular oncology arena now has a dozen new proteomic targets suitable for validation and elucidation of their clinical utility *via* gene knockdown in cellular and animal models. This new method can be replicated and applied to other cancers or disease states for research and development and discovery-based investigations. Our findings, which serve as a proof of concept, will hopefully motivate research groups to further expound on the molecular processes involved in the aggressiveness of EAC and other solid tumor diseases, ultimately leading to improved patient management strategies.

## Introduction

In the last year, our group at Creighton University School of Medicine has reported on the difficulties of the patient management strategies currently utilized in esophageal cancer care (1–3). Indeed, there is an enormous amount of investigation needed in this arena of molecular oncology to better understand the pathogenesis and enhance treatment protocols for esophageal adenocarcinoma (EAC). Noting the demand for novel therapeutic and diagnostic targets, we designed a mass spectrometry-based proteomic study analyzing 50 tissues involved in the progression of EAC. The vast majority of available cancer tissue specimen are fixed with formalin and embedded in paraffin for long-term preservation (4). Formalin-fixed tissue has been typically incompatible with mass spectrometric platforms in the past. However, we utilized a proprietary method known as Liquid Tissue^®^ (Expression Pathology, Inc.) to allow us to analyze protein expression in our new and old formalin-fixed paraffin-embedded (FFPE) tissue samples for retrospective and prospective analysis (4). Utilizing mass spectrometry would allow us to cast a very large proteomic dragnet that could help us see which oncoproteins were being ramped up or turned down during EAC pathogenesis in most, if not all, of our patients.

HER2 and EGFR are two biomarkers that are highly targeted in gastrointestinal cancers with monoclonal antibody-based anticancer therapy. However, these extracellular proteins are expressed in a minority of cases, and drugs that target these markers have had poor performance in esophageal cancer patients (5, 6). Esophageal cancer is also one of the deadliest cancers in America, with its rate of incidence increasing more than any other cancer, while the 5-year survival rate has remained around 12% even during the targeted therapy boom of the last two decades (1). Recently, it was published that adjuvant chemoradiation has no effect on extending survival rates in patients who have undergone esophagectomy surgery for EAC (7). Therefore, we were interested in discovering new tumor drivers overexpressed in the majority of EAC tumors and surrounding tissue to unveil highly involved antagonist drug targets that would have clinical utility in a majority of EAC cases. Conversely, we also wanted to unveil important tumor suppressors that might be turned off during EAC progression, which would yield viable agonist drug targets. Lastly, we hoped to explain why EAC tumors respond poorly to the FDA-approved chemotherapies for esophageal cancer. We therefore utilized a highly conservative biostatistical algorithm and bioinformatics process for our large mass spectrometry data set that delivered us the most statistically significant proteomic events tied to their role in carcinogenesis.

To our knowledge, our investigation in Barrett’s esophageal pathogenesis into EAC was the most extensive mass spectrometry-based experiment utilizing FFPE esophageal tissue reported in scientific literature. From these results, we assert that esophageal cancer tumor tissue derived from Barrett’s esophagus (adjacent precancerous and dysplastic tissue) possesses proteomic expression aberrations which yield an aggressive pathogenesis and resistance to chemotherapeutic agents currently in use as the standard of care. We found 12 highly unique expression patterns which we believe play a role in accelerated proliferation, decreased apoptosis, and tumor suppression as well as chemotherapeutic resistance. We reason that the elucidation of these proteomic abnormalities will yield new drug targets with specific clinical utility for EAC patients while also predicting which Barrett’s esophageal tissue is undergoing carcinogenesis. These newly discovered markers have never been described in peer-reviewed literature as playing a role in any esophageal diseases; therefore, the findings of any further analysis will be original. We propose that additional quantitative and qualitative investigation into these markers will yield multi-avenue prognostic and therapeutic approaches for a field that is in dire need of new drug targets while concurrently revealing why resistance to standard chemotherapies exists in EAC tumors.

## Materials and Methods

**Figure 1:**
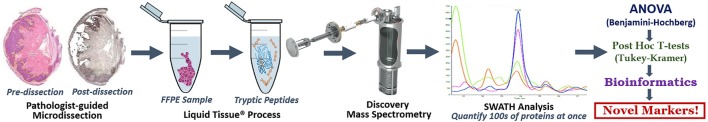
Discovery LT-SWATH-MS Workflow. H&E stained sections of our specimen were marked by a board-certified pathologist and used as a guide for microdissections of serial sections stained with hematoxylin. We aimed to attain 12–15 mm^2^ of pure tumor, Barrett’s esophagus (precancerous tissue) or normal squamous esophageal epithelium in each sample. Pre- and post-microdissection images were taken to ensure that tissue retrieval occurred within the specified margins. The microdissected formalin-fixed paraffin-embedded tissue was placed in Eppendorf tubes and covered in roughly 40 µL of Liquid Tissue^®^ buffer and through a series of heating and digestion stages turned our fixed tissue into a mass spec-friendly lysate. The resultant digest was quantified for protein concentration *via* nanodrop, cleaned *via* MCX, and analyzed by TripleTOF mass spectrometry. SWATH analysis and our outlined series of biostatistics (ANOVA, Benjamini–Hochberg procedure, *post hoc T*-tests, Tukey–Kramer range test), and simple bioinformatics yielded a list of a dozen markers, never before described as being involved in EAC pathogenesis.

This comprehensive protocol covers, end to end, a simple, inexpensive and duplicatable method to discover new markers for solid tumor cancers (Figure 1).

### Ethical Principles for Medical Research Involving Human Subjects

This study was carried out in accordance of (114.1 C) *Exempt Status for review of existing data [per 45CFR46.101 (b) 4] and HIPAA Authorization Waiver*, and (114.1D) *Exempt Status for use of Biological Specimens in Research*. The protocol was examined and approved by the Creighton University Institutional Review Board (Omaha, NE, USA)—IRB# 810702-1. Written informed consent was waived in accordance with the Declaration of Helsinki due to this study’s status of *Existing Data Research* and *Existing De-Identified Specimen Research*. This study used residual specimens that were obtained originally for clinical treatment or pathology purposes independent of this research project.

### Esophagectomy and Tissue Acquisition

After appropriate clinical staging, peri-operative risk assessment, and counseling, the patients underwent esophageal resection. The procedure was completed with esophago-gastric anastomosis, and patients were taken to intensive care unit. Postoperative course was guided by clinical status. While over time, an increasing proportion of patients underwent minimally invasive procedures, yet general principles were adhered to. These included generous En bloc resection of celiac axis and mid/lower thoracic lymph nodes. We also obtained at least 5-cm proximal and distal margins from palpable tumor. After resection, the specimen was “opened” by longitudinally cutting the esophagus/stomach by the pathology resident on a separate non-sterile back table. The proximal and distal margins were visually confirmed, and if needed, a frozen section was obtained. All other remaining specimens were formalin-fixed and later paraffin-embedded for long-term preservation and future pathology-based analysis.

### Patient Selection

We had access to 205 esophagectomy patient specimen collected between 2004 and 2015. Most cases provided multiple blocks of tumor, Barrett’s, and normal esophageal mucosa located in the proximal surgical margins taken from the distal esophagus. Of these 205 patients, 123 of them received neoadjuvant chemotherapy and/or radiation. Due to the destructive effect of chemoradiation, the proteomic milieu of these samples was deemed unreliable. That left us 82 esophagectomy patients with unadulterated tissue untouched by chemoradiation. Of these 82 patients, about a dozen dropped out due to squamous cell carcinoma and gastric cancer histologies. For the mass spectrometry arm of our study we utilized 18 esophageal adenocarcinoma, 7 Barrett's esophagus, and 20 normal squamous mucosa samples, all randomly selected. 15 of the 45 specimens (33%) were from female patients, which reflects the national percentages of gender manifestations for this disease. Ten of the 20 normal esophagus specimen and 9 of the 18 adenocarcinoma specimen were from patients with no visible Barrett's esophagus (50%).

### Pathology Markup and Microdissection

Formalin-fixed paraffin-embedded tissue was cut at 10 µm with one 5-µm section stained with H&E. The paraffin blocks were selected based on the available gross description of the specimens to target normal stratified squamous esophageal mucosa, Barrett’s esophagus, and invasive adenocarcinoma. Histologic slides of areas representing these gross pathologies were examined by a board-certified pathologist, and if histologically confirmed to represent the desired pathology, the area of interest was circled. The serially sectioned unstained slide beneath that examined H&E was then microdissected from the circled area.

Barrett’s esophagus was defined as per the American College of Gastroenterology guidelines as intestinal metaplasia in areas of salmon-colored mucosa in the tubular esophagus (8). Invasive adenocarcinoma areas were selected based on malignant cells clearly infiltrating through the basement membrane into the underlying tissues. Areas of dysplasia, carcinoma *in situ*, or with questionable infiltration were not selected as representative for analysis as either Barrett’s esophagus or invasive adenocarcinoma (9). Normal esophageal mucosa was taken from the proximal resection margin whenever possible to select tissue as far removed from the accompanying pathologic processes. The squamous epithelium with some submucosa was selected for normal mucosa with muscularis propria, and adventitia was not included for analysis.

### Liquid Tissue^®^ Process

Clinically, mass spec platforms typically utilize serum and fresh or frozen tissue specimen for proteomic scrutiny. Formalin cross-linked tissue samples have been found to not completely digest into a lysate compatible for mass spec and therefore increases the risk of breaking expensive machinery. The Liquid Tissue^®^ assay (Expression Pathology, Inc.) has been validated to fully digest FFPE tissue into small, mass spec-friendly peptides. Liquid Tissue^®^ processing eliminates the effects of poor tissue fixation after surgery, as the mass spec measures a small unique identifying peptide within a large protein that only exists in the marker being detected; hence, protein degradation or breakage because of improper fixation has no bearing on the results (4). The liquid tissue process takes about 24 h and completely reverses formalin cross-links of FFPE tissue while eliminating the need for protein epitope integrity for antibody recognition. Lastly, liquid biopsies can be stored indefinitely for future testing.

### SWATH-MS

#### Mass Spectrometry

Data-independent acquisition (DIA) SWATH-MS experiments were performed as described previously (10). All samples were analyzed by reverse-phase high-pressure liquid chromatography electrospray ionization tandem mass spectrometry (RP-HPLC-ESI-MS/MS) using a commercial 6600 TripleTOF^®^ (Sciex) mass spectrometer. The mass spectrometer was coupled with nanoFlex cHiPLC system (Eksigent). All samples were loaded using a stepwise flow rate of 10 µL/min for 8.5 min and 2 µL/min for 1 min using 100% solvent A [0.1% (v/v) formic acid in HPLC water]. Samples were eluted from the analytical column at a flow rate of 0.3 µL/min using a linear gradient of 5% solvent B [acetonitrile with 0.1% (v/v) formic acid] to 35% solvent B over a duration of 180 min. The column was regenerated by washing with 90% solvent B for 15 min and re-equilibrated with 5% solvent B for 15 min. Autocalibration of spectra occurred after acquisition of every six samples using dynamic LC–MS and MS/MS acquisitions of 25 fmol β-galactosidase. Samples used to generate the SWATH-MS spectral library were subjected to traditional, data-dependent acquisition, and library was created using ProteinPilot software v. 4.2 (Sciex). Experimental samples were subjected to cyclic DIA of mass spectra using variable swaths. Ions were fragmented for each MS/MS experiment in the collision cell using rolling collision energy.

#### Targeted Data Extraction

Spectral alignment and targeted data extraction of DIA samples were performed using PeakView v.1.2 (Sciex) using the MDM reference spectral library generated above. All DIA files were loaded and exported in.txt format in unison using an extraction window of 10 min and the following parameters: 5 peptides, 5 transitions, peptide confidence of >99%, exclude shared peptides, and XIC width set at 50 ppm. This export results in the generation of three distinct files containing the quantitative output for (1) the area under the intensity curve for individual ions, (2) the summed intensity of individual ions for a given peptide, and (3) the summed intensity of peptides for a given protein. Laboratory contaminants and reversed sequences were removed from the data set prior to statistical analysis.

### Biostatistics

A total of 617 proteins were identified and quantified by mass spectroscopy-SWATH analysis. Mass spectrometric values were converted to log base 2 prior to statistical analysis. One-way fixed effect ANOVA was used to detect differences between the three tissue types (normal squamous esophageal epithelium, Barrett’s esophagus, and adenocarcinoma tumor tissue). The Benjamini–Hochberg procedure was used to determine which of the ANOVA tests indicated a detectable association between protein expression and tissue type while controlling the false discovery rate (FDR) at no more than 0.05. The *post hoc T*-tests were allotted an alpha of 0.00085 and adjusted using the Tukey–Kramer adjustment for multiple comparisons. All of our calculations were processed with SAS 9.4.

### Bioinformatics

After our biostatistics workflow, our proteomic data set whittled down from 1,100 involved proteins to a 164 potential protumorigenic suspects based upon the significant difference between expression levels of normal, Barrett’s, and EAC tissue. These proteins, identified and quantified *via* discovery mass spectrometry, were ordered by *P*-value and biomarkers with *P*-values less than 0.0001 and were also below the FDR considered for further analysis (Benjamini–Hochberg). We then went one by one through each marker to determine their role in oncological processes and entered this information into a spreadsheet, assigning a score to each protein: III—unequivocal role in cellular proliferation and tumor suppression, II—semi-indicative of involvement in cancer, I—no intersections with molecular oncology. After this process, we had 20 markers with a score of III, 58 markers with a score of II, and 86 markers with a score of I. Of the 20 III-score markers, 12 are listed in this paper. Using several different databases (i.e., genecards and uniprot), the general gene functions of the affiliated proteins were listed. This allowed us to identify common pathways that were changing during the oncogenic transformation from normal tissue to Barrett’s tissue to EAC tissue. In addition, the classification of the markers allowed us to identify a group of highly impactful genes that had been previously described to contribute to carcinogenesis and resistance in many cancers but had yet to be described in EAC. To focus our potential targets, we referred to the DrugBank database (version 5.0) which identified previously described protein and drug interactions. We considered the common first-line chemotherapy drugs for this cancer; cisplatin, 5-FU, and taxol to integrate our proteomic data with markers associated with chemoresistance. This workflow allowed us to identify high-yield novel biomarkers that are known to contribute to therapy resistance in other cancers and are likely contribute to the disease progression and observed chemoresistance in EAC.

## Results

See Table 1 for a summary of our proteomic expression patterns, biostatistics, and the prognostic, diagnostic, and therapeutic utility of our findings.

**Table 1:** Summary of results: details of the 12 novel markers found to be involved in the pathogenesis of EAC.

**Novel marker**	**Role in EAC progression**	**Expression difference tumor vs normal**	**Tumor vs Normal expression (*P*-value)**	**Specimen with similar expression patterns (Figure 4)**	**Described role as an oncoprotein in other cancers**	**Drug target type**	**Chemoresistance**
ANXA1 (Annexin A1)	Decreases Tumor Suppression	−8.75	1.67E-15	20 out of 20	Downregulation associated with more rapid cancer recurrence (6 months vs 2 years) in bladder cancer (11)	Potential agonist	Downregulation associated with resistance to chemoradiation (11, 12)
CRNN (Cornulin)	Decreases Tumor Suppression	−34.28	0.00	20 out of 20	Downregulation associated with greater tumor length, tumor invasion and lymph node metastasis and lower survival in ESCC (13)	Potential agonist	No evidence found in the literature
HMGB1 (Amphoterin)	Increases Cellular Proliferation	2.33	1.23E-05	19 out of 20	Overexpression associated with poorer prognosis in CRC patients. Inhibition prolonged survival in mesothelioma mice model (14, 15)	Potential antagonist	Overexpression associated with resistance to cisplatin in lung cancer (16)
IL-1RA	Decreases Tumor Suppression	−15.34	2.22E-16	20 out of 20	IL-1RA disrupts IL-1 from playing a role in tumor development and progression and demonstrated antitumor activity in melanoma (17)	Already an antagonist	Downregulation associated with resistance to cisplatin and docetaxel (17)
KRT7 (Keratin 7)	Increases Cellular Proliferation	11.67	5.56E-08	20 out of 20	Overexpression associated with higher morbidity and higher progression in CRC (18)	Potential antagonist	No evidence found in the literature
LGALS3BP (Galectin-3)	Increases Cellular Proliferation	3.50	3.51E-05	20 out of 20	Breast and lung cancer cells overexpressing LGALS3BP show apoptosis resistance in response to cisplatin (19, 20)	Potential antagonist	Overexpression associated with resistance to cisplatin (20)
LTF (Lactoferen)	Increases Cellular Proliferation	9.56	2.65E-06	18 out of 20	Overexpression associated with migration and proliferation in nasopharyngeal carcinoma and endometrial cancer (21, 22)	Potential antagonist	No evidence found in the literature
PRMT1	Increases Cellular Proliferation	2.02	6.50E-05	19 out of 20	Nuclear expression is associated with poor prognosis and chemoresistance in gastric cancer. Upregulated in NSCLC (23–25)	Potential antagonist	Overexpression associated with resistance to cisplatin and 5-FU (23)
S100A8	Decreases Tumor Suppression	−7.75	1.98E-09	20 out of 20	Downregulation is associated with poor prognosis and low rates of survival in head and neck squamous cell carcinoma (26)	Potential agonist	Downregulation associated with resistance to cisplatin and paclitaxel (27)
S100P	Increases Cellular Proliferation	7.22	6.05E-08	19 out of 20	Expression increases is associated w/ poor prognosis and shorter survival in gastric, pancreatic and ovarian cancer (28, 29)	Potential antagonist	Overexpression associated with resistance to 5-fluorouracil (28)
SFN (14-3-3 Sigma)	Decreases Tumor Suppression	−10.49	1.01E-13	20 out of 20	Downregulation correlates with multistage carcinogenesis and poor prognosis in ESCC/ salivary gland adenoid cystic cancer (30, 31)	Potential agonist	Downregulation associated with resistance to cisplatin (32)
TXN (Thioredoxin)	Decreases Tumor Suppression	−2.07	5.96E-06	19 out of 20	Downregulation in lung cancer results in increased ROS and alteration in tumor metabolism resulting in cisplatin resistance (33)	Potential antagonist	Downregulation associated with resistance to cisplatin (33)

*The table includes the expression difference values between normal and tumor cells, the percentage of patients exhibiting these expression patterns, the descriptions of these markers involvement in other cancerous indications, potential drug types to target these markers, and reported associations to chemoresistance (12, 15, 16, 21, 25–27, 32, 34). 5-FU, fluorouracil; CRC, colorectal cancer; ESCC, esophageal squamous cell carcinoma; NSCLC, non-small cell lung carcinoma*.

### Proliferation Markers (Prognostic)

#### HMGB1

**Figure 2:**
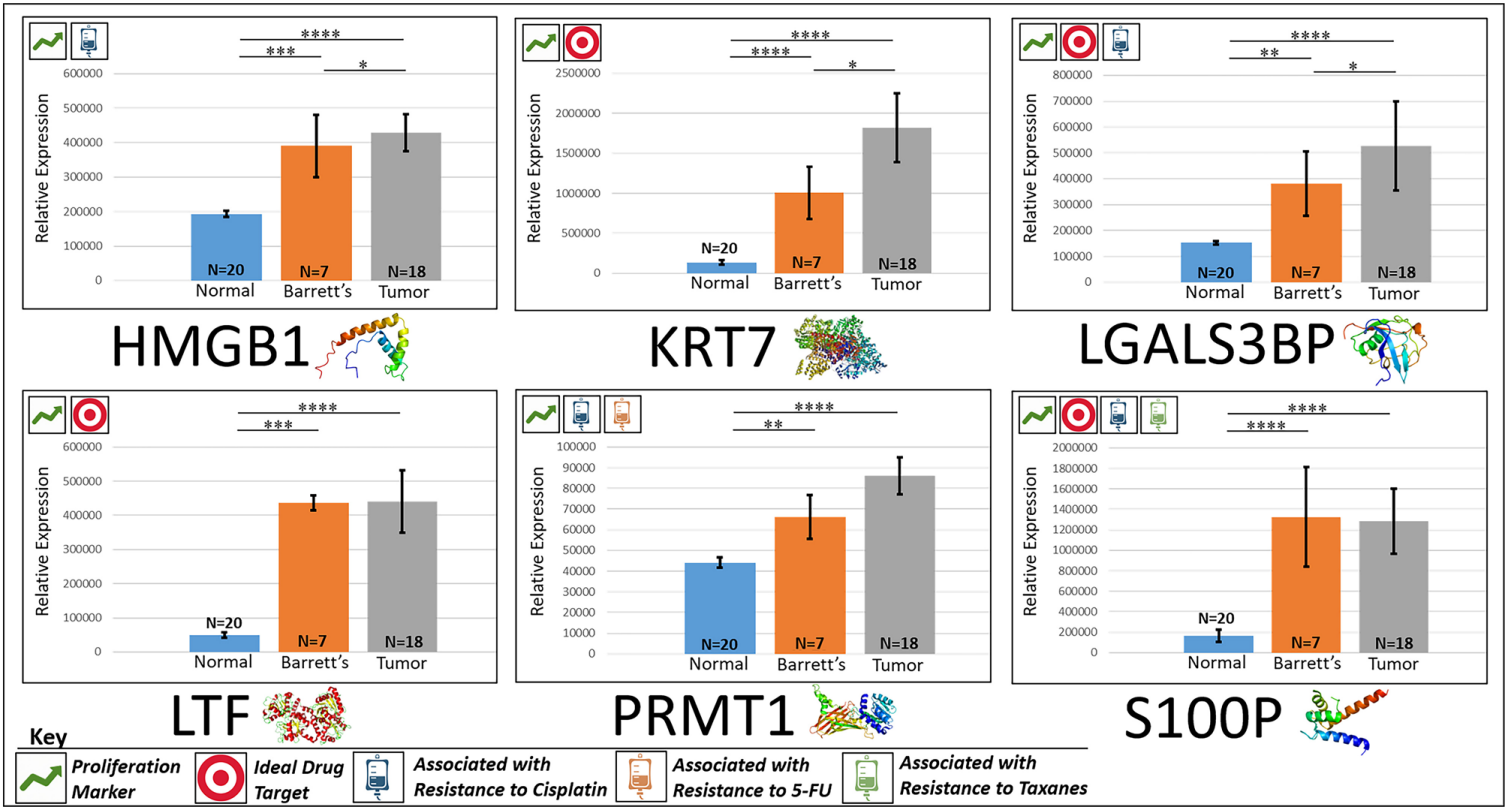
Upregulated proliferation markers. These six markers are associated with more advanced progression in various cancers and were found to be upregulated or overexpressed in our cohort’s esophageal adenocarcinoma tumor tissue compared to normal squamous esophageal epithelium, possibly contributing to enhanced invasiveness and shorter overall survival. ****P < 0.0001, ***P < 0.001, **P < 0.01, *P < 0.1.

HMGB1, or high mobility group box 1, is located in the nucleus and is one of the major chromatin-associated non-histone proteins, acting as a DNA chaperone involved in replication, transcription, chromatin remodeling, and DNA repair (35) (for expression patterns of all six proteins, see Figure 2). Treatment with HMGB1 inhibitors prolonged the survival of malignant mesothelioma xenograft mice (14). HMGB1 overexpression is associated with poorer prognosis in colorectal cancer patients (15). HMGB1 was expressed 2.33× more in EAC tumors compared to the adjacent normal esophagus epithelium according to our findings (*P* < 0.0001).

#### KRT7

KRT7, or keratin 7, stimulates DNA synthesis in several cell types. Aberrant expression of KRT7 in budding cancer cells represents a modification of the epithelial phenotype (epithelial–epithelial transition) which may be linked to gains in motility and invasive potential (18). KRT7 expression is associated with a higher morbidity and a higher progression in colorectal cancer (18). KRT7 is overexpressed 11.67× more in EAC tumors compared to normal esophageal tissue according to our findings (*P* < 0.0001).

#### LGALS3BP

LGALS3BP, or Galectin-3-binding protein, promotes integrin-mediated cell adhesion associated with cancer. This protein has a high affinity for beta-galactoside and has been found to be expressed in many tumor cells associated with aggressive carcinogenesis (19). Breast and lung cancer cells overexpressing LGALS3BP demonstrated resistance to apoptosis in response to cisplatin (20). We found that LGALS3BP is overexpressed at a 3.50× greater level in EAC tumor cells compared to normal esophageal cells (*P* < 0.0001).

#### LTF

LTF, or lactotransferrin, stimulates the TLR4-signaling pathway, leading to NFkβ activation and subsequent pro-inflammatory cytokine production while also interfering with the lipopolysaccharide-stimulated TLR4 signaling and also stimulates VEGF-mediated endothelial cell migration and proliferation (21). Malignant transformation of endometrial tissue is associated with overexpressed LTF (22). LTF is expressed at a 9.56× greater level in EAC tumors compared to normal esophageal tissue according to our findings (*P* < 0.0001).

#### PRMT1

PRMT1 is the main enzyme that mediates the methylation of histone H4, a specific tag for epigenetic transcriptional activation. PRMT1 has also been identified as a key regulator of the epithelial–mesenchymal transition in breast cancer (36). PRMT1 expression is associated with poor prognosis in gastric cancer patients and has been observed to be significantly upregulated in non-small cell lung carcinoma (23, 24). Knockdown of PRMT1 in three NSCLC cell lines was associated with a significant suppression of cell growth (25). We found PRMT1 to be expressed at a 2.02× greater level in EAC tumor cells compared to normal esophageal epithelial cells (*P* < 0.0001).

#### S100P

S100P proteins are localized in the cytoplasm of a wide range of cells and involved in the regulation of several cellular processes such as cell cycle progression and differentiation (34). Significant correlation was found between high expression and S100P and shorter overall survival (OS) and increased drug resistance in gastric and ovarian cancer (28). S100P also plays a key role in the aggressiveness of pancreatic cancer which is likely mediated by its ability to activate RAGE (29). We found S100P to be expressed at a 7.22× greater level in EAC tumors compared to normal esophagus tissue (*P* < 0.0001). A heat map of the six upregulated tumor drivers and their expression patterns during esophageal pathogenesis can be found in Figure 4.

### Silenced “Good Guys” (Prognostic)

#### ANXA1

**Figure 3:**
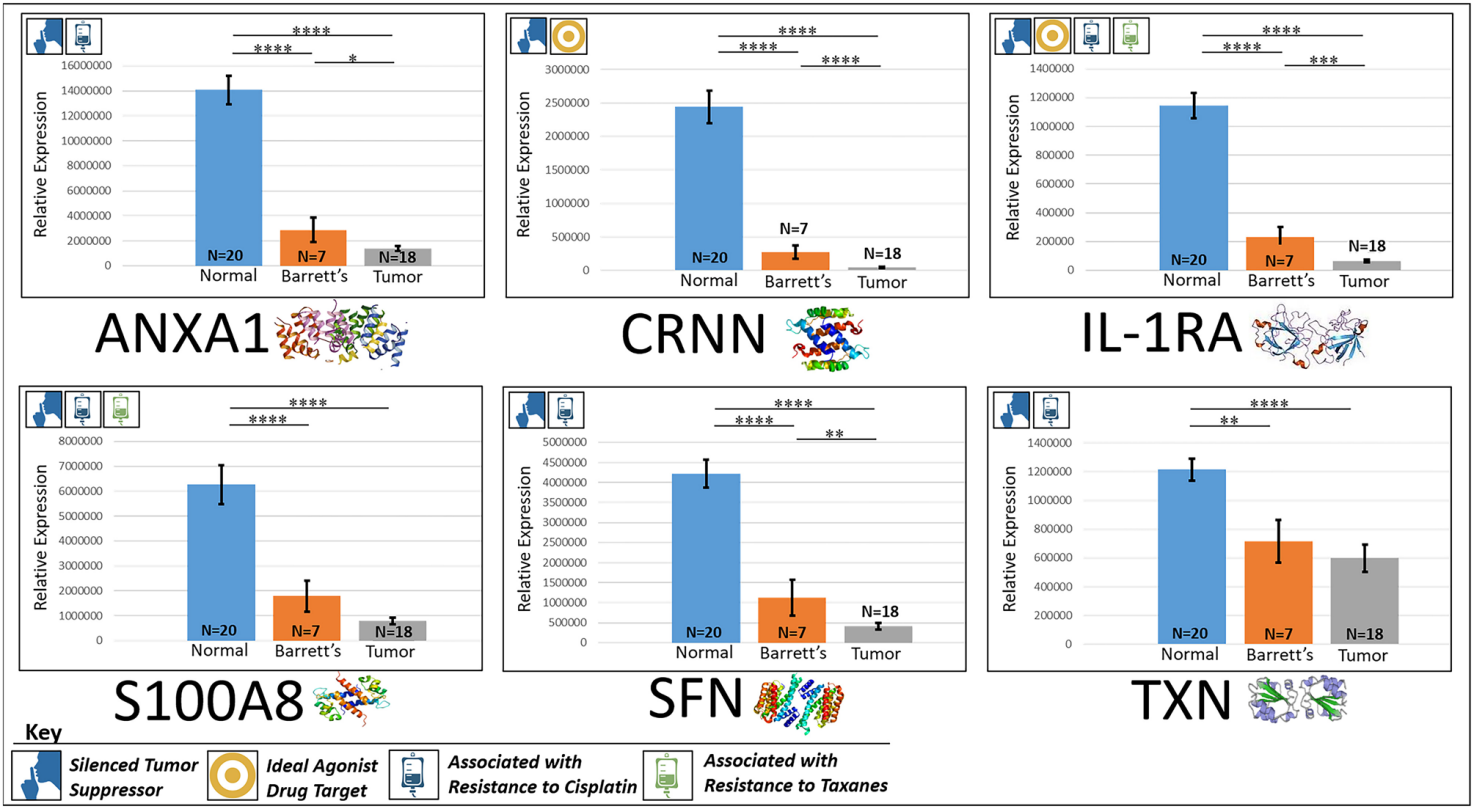
Downregulated tumor suppressors. These six markers are associated with inducing programmed cell death in various cancers and were found to be downregulated in our cohort’s esophageal adenocarcinoma tumor tissue compared to normal squamous esophageal epithelium, possibly contributing to enhanced invasiveness and shorter overall survival. ****P < 0.0001, ***P < 0.001, **P < 0.01, *P < 0.1.

ANXA1, or annexin A1, has anti-inflammatory activity and contributes to the adaptive immune response by enhancing signaling cascades that are triggered by T-cell activation (11) (for expression patterns of all six proteins, see Figure 3). Downregulation of ANXA1 is associated with more rapid cancer recurrence in bladder cancer (6 months vs 12 years) (11). Knockdown of ANXA1 was found to block the intake of chemotherapy, leading to anticancer drug resistance (11). Downregulation of ANXA1 has also been associated with radiotherapy resistance and increased relapse rates in head and neck cancer (12). ANXA1 is expressed −8.75× less in EAC tumors compared to normal esophageal tissue exhibiting massive downregulation (*P* < 0.0001).

#### CRNN

CRNN, or cornulin, is a survival factor that participates in the proliferation of squamous esophageal epithelial cells and attenuates apoptotic cell death. Heat shock proteins like CRNN play a critical role in controlling unusual environmental pressures placed on squamous epithelial cells (37). Loss of CRNN expression has been correlated with an advanced tumor length, a greater tumor invasion depth, lymph node metastasis, and poor survival in patients with esophageal squamous cell carcinoma (13). On the other hand, patients with high CRNN gene expression were more likely to achieve a pathologic complete response to neoadjuvant chemoradiotherapy (13). However, CRNN is expressed −34.28× less in EAC tumor cells compared to normal esophageal epithelial cells according to our findings (*P* < 0.0001), adding CRNN as a viable oncoprotein for EAC as well.

#### IL-1RA

IL-1RA is an endogenous protein that inhibits the activity of interleukin-1 by binding to receptor IL-1R and preventing its association with its co-receptor for signaling (17). Multiple studies indicate that IL-1 plays a role in tumor development and progression, and a high expression of IL-1RA is associated with antitumor activity in various cancer models, including melanoma (17). Also, patients receiving IL-1RA prior to chemotherapy were found to have enhanced treatment responses (17). We found IL-1RA to be expressed −15.34× less in EAC tumors compared to normal esophageal tissue (*P* < 0.0001).

#### S100A8

S100A8 has extracellular functions involved in pro-inflammatory, antimicrobial, and apoptosis-inducing activities (38). Calprotectin, a heterodimeric complex of the calcium-binding proteins S100A8 and S100A9, regulates cell cycle progression at G2/M, inhibiting cancer cell migration and invasion, and suppressing tumorigenesis *in vitro* and *in vivo* (26). Downregulation of S100A8 in head and neck squamous cell carcinoma is associated with poor prognosis and lower rates of survival (26). And on the other hand, a high S100A8 expression was found to be a favorable prognostic factor for the survival of oropharyngeal squamous cell carcinoma (38). We found S100A8 to be expressed −7.75× less in EAC tumor cells compared to normal epithelial cells of the esophagus (*P* < 0.0001).

#### SFN

SFN, or 14-3-3 sigma, is an adapter protein involved in regulating both general and specialized signaling pathways. Downregulation of SFN has been associated with multistage carcinogenesis and poor prognosis in salivary gland adenoid cystic carcinoma and esophageal squamous cell carcinoma (30, 31). We found SFN to be expressed −10.49× less in EAC tumor tissue compared to normal esophagus (*P* < 0.0001).

#### TXN

**Figure 4:**
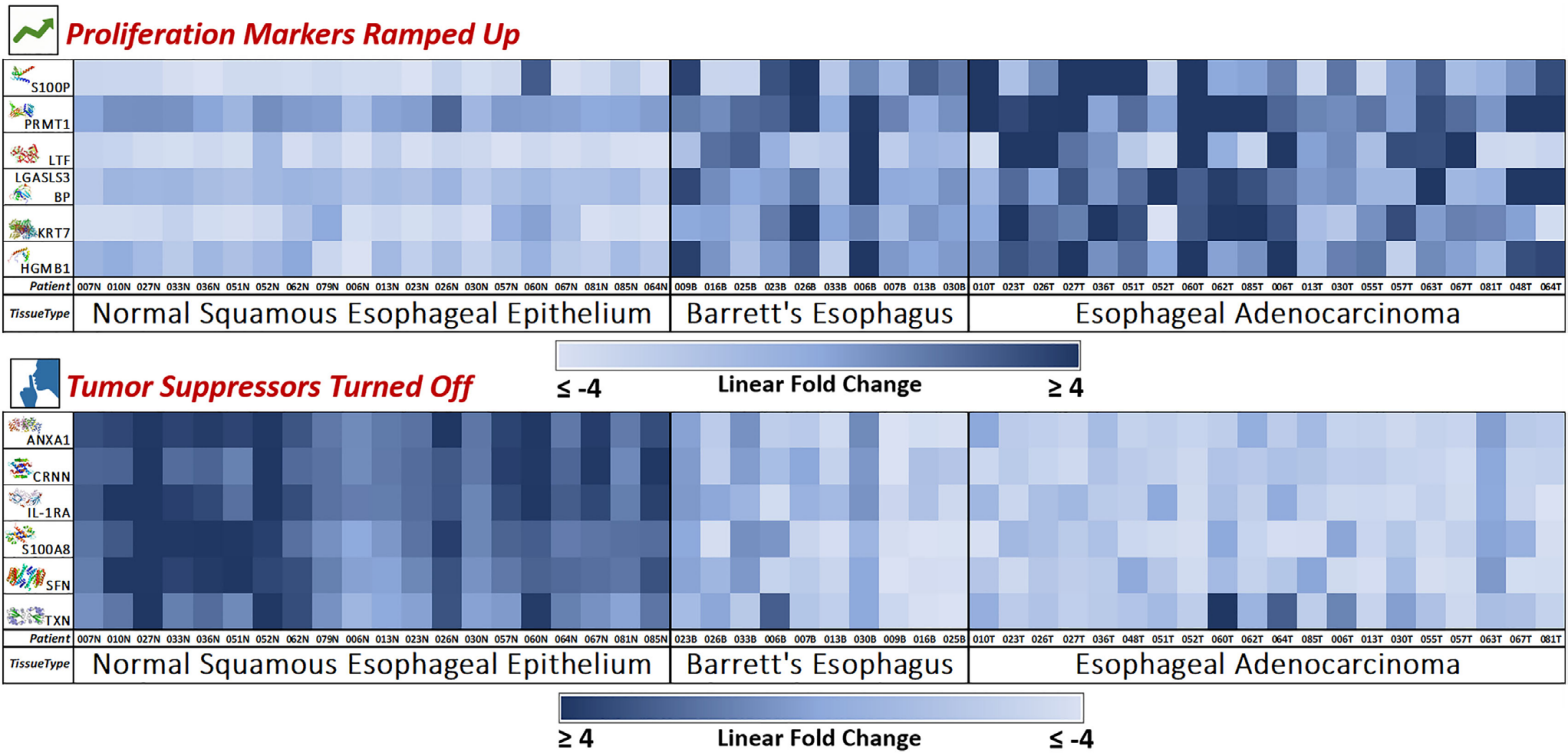
Novel marker heat maps—proliferation up, tumor suppression down. Heat maps of the two groups of novel markers (cellular proliferation and tumor suppression) found to be upregulated or downregulated during esophageal pathogenesis (Normal → Barrett’s → Adenocarcinoma). This provides for an individualized bird’s eye view of the expression trends of our newly discovered 12 markers in 50 patient samples, showing consistent trends with very few outliers. Here, we see how the mass spectrometry platform can yield statistically significant biomarkers with consistent and reliable expression trends. Notably, Barrett’s tissue shows expression levels similar to adenocarcinoma, meaning that these protumoral expression trends manifest in the precancerous Barrett’s tissue and carries over throughout carcinogenesis.

TXN, or thioredoxin, participates in various redox reactions and catalyzes dithiol–disulfide exchange reactions. Downregulation of TXN in lung cancer results in an increased reactive oxygen species and alters tumor metabolism, resulting in cisplatin resistance (33). We found TXN to be expressed −2.07× less in EAC tumors compared to normal esophageal tissue (*P* < 0.0001). A heat map of the six downregulated tumor suppressors and their expression patterns during esophageal pathogenesis can be found in Figure 4.

### New Drug Targets (Therapeutic)

CRNN, IL-1RA, KRT7, LGALs3BP, LTF, and S100P are viable targets for EAC anticancer therapy. Five of the six markers possess suitable binding pockets as well as auxiliary pockets for monoclonal antibody targeted therapy. S100P, on the other hand, could be targeted *via* RAGE protein which would serve as an inhibitor to S100P’s contribution to esophageal cancer’s overall progression. CRNN would be considered an agonist therapeutic target, where upregulating CRNN *via* drug agonism could potentially induce apoptosis in EAC tumors. IL-1RA in its natural form is already an antagonist, where simply treating with endogenous IL-1RA would block IL-1 and presumably yield favorable anticancer effects.

#### CRNN

CRNN is a potential target for agonist therapy. It possesses suitable binding pocket as well as an auxiliary pocket for monoclonal targeted therapy. Increasing CRNN levels *via* drug agonism in EAC tumors could potentially decrease tumor length, minimize tumor invasion, decrease lymph node metastasis, and increase survival.

#### IL-1RA

IL-1RA is a potential molecule for antagonist therapy. Injecting an EAC tumor with IL-1RA would block IL-1R, leading to the deactivation of IL-1. This series of events would presumably lead to more favorable anticancer conditions such as a decreased proliferation and an increased apoptosis.

#### KRT7

KRT7 is a potential target for antagonist therapy. KRT7 possesses the proper binding pocket for monoclonal antibody attachment, and inhibiting this protein in an EAC patient would potentially lead to more favorable anticancer conditions such as a decreased cellular proliferation.

#### LGALS3BP

LGALS3BP is a potential target for antagonist therapy and possesses a suitable binding pocket for drug action. Inhibiting LGALS3BP could presumably stimulate the adaptive immune system against EAC tissue while also increasing sensitivity to cisplatin.

#### LTF

LTF is a potential target for antagonist therapy and has a favorable binding pocket for antibody attachment. Blocking LTF could potentially inhibit epithelial proliferation and tumorigenesis in EAC.

#### S100P

S100P is a potential target for antagonist therapy; however, this protein does not possess the proper binding pocket to allow for therapeutic action. Therefore, a viable route to block S100P action is *via* RAGE inhibition. S100P-derived RAGE antagonistic peptide has been found to delay tumor growth and metastasis in pancreatic cancer (39).

### Chemoresistance Markers (Diagnostic)

It was recently published that chemotherapy has no effect in patients with loco-regional or advanced esophageal cancers (7). Advanced esophageal cancer patients who received chemotherapy had no survival advantage compared to patients who received no treatment course at all (7). About 95% of EAC patients are prescribed cisplatin in the first-line setting (3). We discovered that seven proteomic expression trends associated with cisplatin resistance were seen to be either overexpressed or downregulated in the vast majority of EAC tumors analyzed in our study—HMGB1, IL-1RA, LGALS3BP, PRMT1, S100A8, SFN, and TXN (11, 12, 16, 17, 20, 23, 27, 28, 32, 33). About 63% of EAC patients receive the chemotherapy drug 5-FU in the first-line setting (3). We discovered two biomarkers associated with 5-FU resistance in other solid tumor cancers that were upregulated in Barrett’s and EAC tissue (S100P, PRMT1), posing a potential multifaceted resistance to 5-FU in precancerous and cancerous esophageal tissue (23, 28). Lastly, we found three markers associated with sensitivity to taxanes downregulated in both Barrett’s and EAC tissue, posing a subsequent resistance to this family of chemotherapies. About 37% of patients receive a taxane as part of their first-line regimen (3). Downregulation of S100A8, IL-IRA, and ANXA1 has been associated with a reduced likelihood of benefit when using taxanes (11, 12, 17, 27). Again, chemoradiation has been found to have an insufficient clinical effect on EAC patients; therefore, these data potentially explains why FDA-approved chemotherapies for esophageal cancer (i.e., cisplatin, 5-FU, and taxanes) have a limited effect on patient response and OS (3, 40). These findings will hopefully prompt a discussion around altering patient management strategies away from the use of cisplatin and other chemotherapies in esophageal cancer care.

## Discussion

Formalin-fixed paraffin-embedded tissue archives and their associated diagnostic records represent an invaluable source of proteomic information on diseases where the patient outcomes are already known. Our approach makes it possible to recover peptides from FFPE tissues that yield a reasonable representation of the proteins recovered from identical fresh or frozen specimens (4). This method allows full spectrum discovery quantitative analysis of the proteome of FFPE archival tissues. As a tertiary referral center for esophageal cancer patients being treated with esophagectomy surgery, our medical center (CUMC) has access to hundreds of esophageal tumors and their adjacent Barrett’s and normal esophageal tissue. Along with these tissues, we have robust pathology and patient history reports, adjuvant therapy strategies, and in some cases, survival statistics. Because there is no clear understanding of how carcinogenesis occurs in Barrett’s esophagus, we utilized an assay or an instrument that allowed us to cast a wide net diagnostically as there could be just a few proteins out of 1,000s that are responsible for this disease progression. Therefore, we opted to utilize the power of mass spectrometric technology which measures all peptides in each sample by their mass and charge, utilizing the laws of physics rather than depending on antibodies which can be susceptible to nonspecific binding or subjective variability. This is a “multiplexable” platform that can simultaneously measure numerous analytes (1,000s) in a single run, yielding false-positive-free and precise quantitative data from only 8–12 mm^2^ of FFPE tissue.

There are 25,000 different types of proteins in each cell. However, only a small number of proteins would be viable players in the carcinogenesis of Barrett’s esophagus. To be able to narrow down this number, we created a protein library from the three different tissue types. We detected ~1,100 different proteins in over two dozen normal esophageal epithelium, Barrett’s esophagus, and EAC tissue samples. From there, we were able to reliably quantify 617 proteins from 50 tissue samples utilizing SWATH data analysis (see Bioinformatics). Quantification readings (femtomole/μg) of 617 proteins from all 50 samples yielded an enormous amount of data. Therefore, we manufactured a conservative biostatistical algorithm and a straightforward bioinformatics procedure to determine which markers would be worth additional investigation. Using an ANOVA, followed by Tukey–Kramer *post hoc T*-test and then a Benjamini–Hochberg procedure to eliminate additional false discoveries, we narrowed down the number of significant proteomic events from 617 to 164. Additional bioinformatics decreased the 164 suspects to 12 statistically significant proteomic events, which potentially play a role in the pathogenesis and progression in most EAC cases. This quick, inexpensive, reproducible new method of analyzing FFPE tissue using a discovery mass spec platform (TripleTOF^®^ 6600—Sciex) yielded a dozen new markers, never described as being involved with EAC, that have significant prognostic, diagnostic, and therapeutic potential.

## Future Studies and Potential Clinical Applications

Our group has begun to validate and elucidate the clinical utility of a number of these markers in human, Yucatan microswine, and cell line models. We are confirming expression trends with alternate proteomic quantification methods and determining the effect of cellular proliferation, migration, and invasion rates using siRNA gene knockdowns of a few of the markers unearthed by this method. So far, our results have been confirmatory and promising, demonstrating that Liquid Tissue-SWATH analysis brought forth new prognostic, therapeutic, and diagnostic markers in an oncology field desperate for new targets. The findings of these validation studies will be submitted for publication this year. We also have designed schemes to retroactively investigate the effect of overexpression of our discovered markers linked to chemotherapeutic resistance in terms of overall and disease-free survival in our 200+ patient cohorts and expound on the biochemical mechanisms at play to see if they are contributing to the ineffectiveness of cisplatin, taxanes, and 5-FU in arresting EAC progression. We also would like to encourage the greater molecular oncology community to not only utilize this method to discover new markers in their solid tumor indication of interest but to also embrace the markers listed herein to further illuminate their role in EAC disease and progression. It was important to us to publish this method and the subsequent findings, which serve as a proof of concept, in an open-access journal with the hope that it will inspire other research groups to further expound on the molecular processes involved in the aggressiveness of EAC and other solid tumors.

## Data Availability

We have made available two files of supplemental data on the Figshare online digital repository. The two files contain (1) raw data from our mass spec proteomics analysis of 50 tissues involved in the pathogenesis of EAC and (2) full biostatistics of the 12 novel markers presented herein. These files can be found at (https://figshare.com/s/c5ec42d6b2cbd9a77405). We have redacted eight markers from the mass spec raw data file as their identities, and corresponding expression trends are included in a provisional patent that was filed by our group and Creighton University through the United States Patent Office on February 6th, 2018 (application no. 62/621724). The diagnostic and therapeutic utility of these eight redacted markers have not been mentioned within this manuscript; however, if you would like to request permission to make these markers and their quantitative data available for non-commercial use, please contact the corresponding author Dr. Devendra Agrawal.

## Author Contributions

JA wrote the first draft of the manuscript, with exception of the method sections. JA designed and created all five figures, created Table 1, managed the mass spectrometry project, performed the majority of the benchwork, and contributed to multiple revisions of the paper. CW performed biostatistics for this study and wrote Biostats subsection in the methods. ND provided a board-certified pathological review of all of our H&E specimens and wrote the microdissection subsection. PC ran our mass spec samples, provided us with a robust data set, consulted on proteomic aspects of our study, and wrote the MS-SWATH subsection in the methods. JF performed benchwork, designed our bioinformatics platform, analyzed our data, designed the figures, and wrote the bioinformatics subsection. SM brought the concept of this study to our group, provided all of the clinical specimens, wrote the Tissue Retrieval subsection, and provided multiple revisions. All of the benchwork was performed in the laboratory of DA. DA contributed to the design of the study, supported the investigation with funding, provided multiple revisions of the manuscript and figures, and supervised the progress of the project over the last two and a half years.

## Conflict of Interest Statement

The authors declare that the research was conducted in the absence of any commercial or financial relationships that could be construed as a potential conflict of interest.
