# Corrigendum: Discovery of Novel and Clinically Relevant Markers in Formalin-Fixed Paraffin-Embedded Esophageal Cancer Specimen

**DOI:** 10.3389/fonc.2019.00273

**Published:** 2019-04-12

**Authors:** Joe Abdo, Christopher S. Wichman, Nicholas E. Dietz, Pawel Ciborowski, John Fleegel, Sumeet K. Mittal, Devendra K. Agrawal

**Affiliations:** ^1^Department of Clinical and Translational Science, Creighton University School of Medicine, Omaha, NE, United States; ^2^Department of Biostatistics, College of Public Health, University of Nebraska Medical Center, Omaha, NE, United States; ^3^Department of Pathology, CHI Health Creighton University Medical Center, College of Medicine, Omaha, NE, United States; ^4^Department of Pharmacology, University of Nebraska Medical Center, Omaha, NE, United States; ^5^Norton Thoracic Institute, St. Joseph's Hospital and Medical Center, Dignity Health, Phoenix, AZ, United States

**Keywords:** mass spectrometry, formalin-fixed paraffin-embedded tissue, SWATH analysis, proliferation markers, proteomics, molecular oncology, chemoresistance markers

In the original article, there was a mistake in Figure 2 and Figure 3 as published. The tissue sample N's were reported as (Normal = 20, Barrett's = 10, Tumor = 20), the actual N's analyzed were (Normal = 20, Barrett's = 7, Tumor = 18). The units for the mass spec quantification data in the Figure 2 and Figure 3 were listed as femtomol per microgram (fmol/μg), however, it should have been classified as “Relative Expression.” The corrected Figure 2 and Figure 3 appears below.

**Figure 2:**
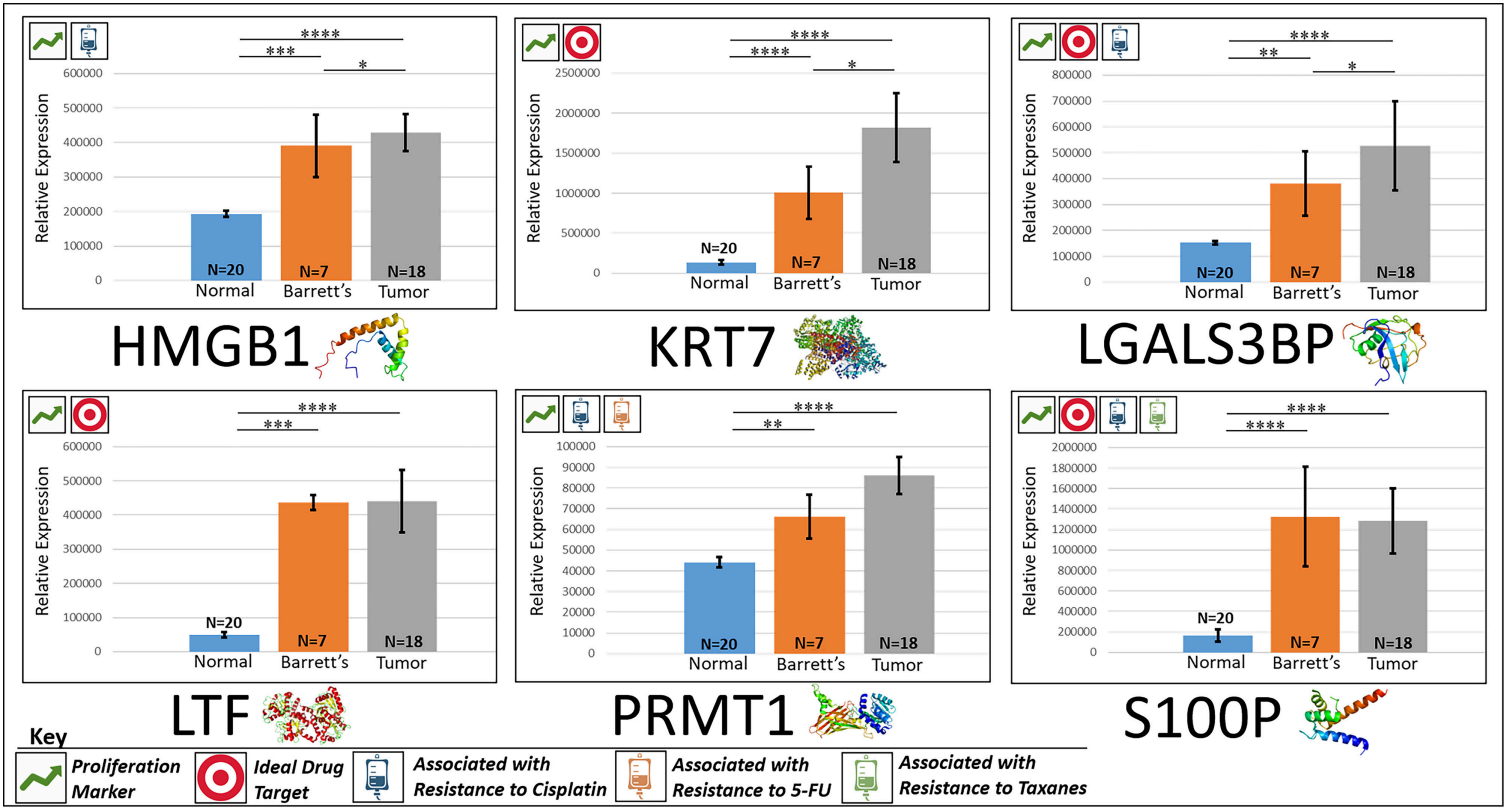
Upregulated proliferation markers. These six markers are associated with more advanced progression in various cancers and were found to be upregulated or overexpressed in our cohort's esophageal adenocarcinoma tumor tissue compared to normal squamous esophageal epithelium, possibly contributing to enhanced invasiveness and shorter overall survival. ****P < 0.0001, ***P < 0.001, **P < 0.01, *P < 0.1.

**Figure 3:**
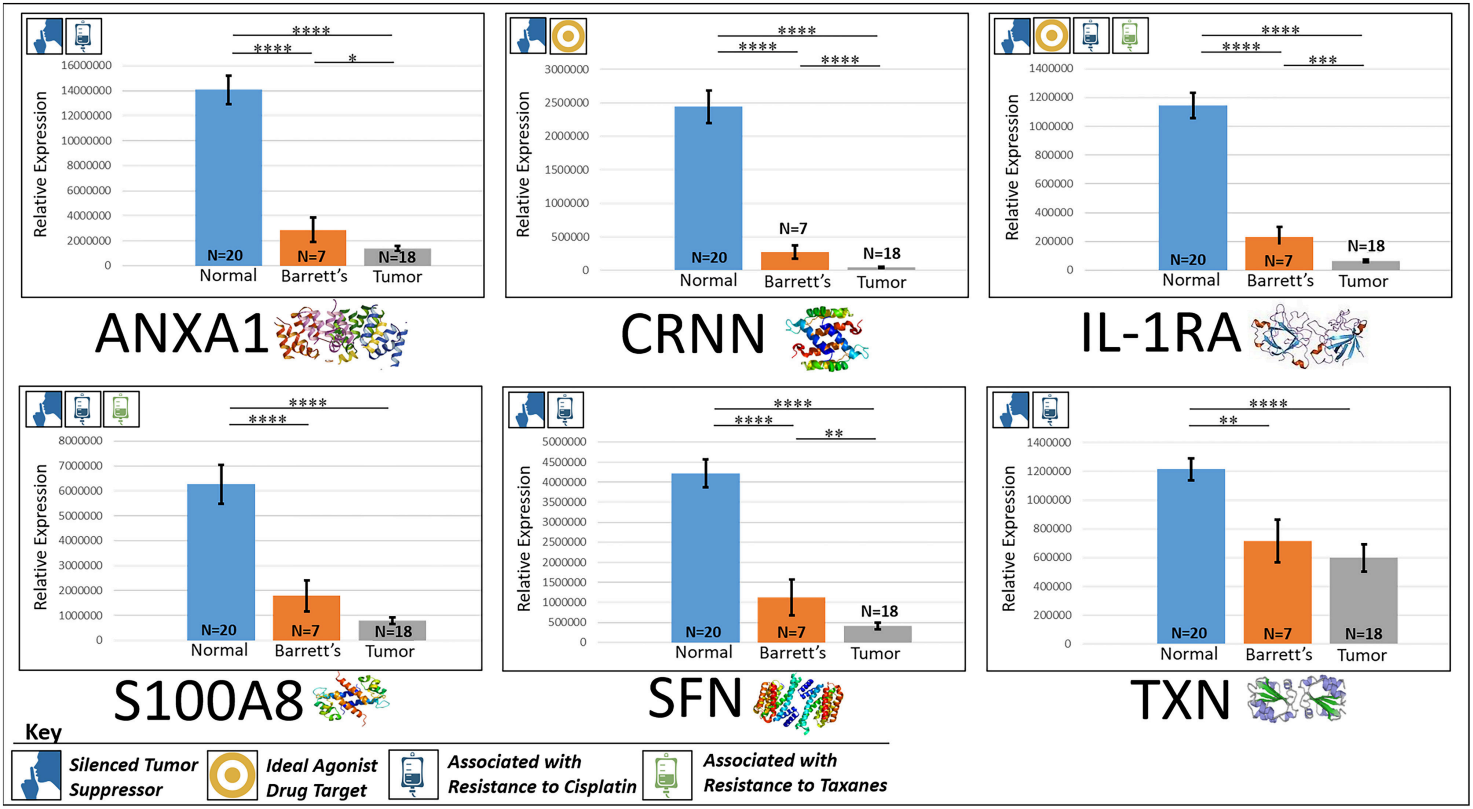
Downregulated tumor suppressors. These six markers are associated with inducing programmed cell death in various cancers and were found to be downregulated in our cohort's esophageal adenocarcinoma tumor tissue compared to normal squamous esophageal epithelium, possibly contributing to enhanced invasiveness and shorter overall survival. ****P < 0.0001, ***P < 0.001, **P < 0.01, *P < 0.1.

Additionally, in the introduction it was reported that 50 tissues were analyzed via mass spectrometry. Forty-five tissue samples were used for the mass spectrometry quantification. In the results section, T-scores were reported rather than the magnitude of expression differences between the normal and esophageal adenocarcinoma tissue in all 12 reported markers of interest.

A correction has been made to the **Materials and Methods**, subsection **Patient Selection**:

“For the mass spectrometry arm of our study we utilized 18 esophageal adenocarcinoma, 7 Barrett's esophagus, and 20 normal squamous mucosa samples, all randomly selected. 15 of the 45 specimens (33%) were from female patients, which reflects the national percentages of gender manifestations for this disease. Ten of the 20 normal esophagus specimen and 9 of the 18 adenocarcinoma specimen were from patients with no visible Barrett's esophagus (50%).”

Furthermore, corrections have been made to the **Results**, subsection **Proliferation Markers (Prognostic)**:

**HMGB1**

“HMGB1, or high mobility group box 1, is located in the nucleus and is one of the major chromatin-associated non-histone proteins, acting as a DNA chaperone involved in replication, transcription, chromatin remodeling, and DNA repair (35) (for expression patterns of all six proteins, see Figure 2). Treatment with HMGB1 inhibitors prolonged the survival of malignant mesothelioma xenograft mice (14). HMGB1 overexpression is associated with poorer prognosis in colorectal cancer patients (15). HMGB1 was expressed 2.33x more in EAC tumors compared to the adjacent normal esophagus epithelium according to our findings (*P* < 0.0001).”

**KRT7**

“KRT7, or keratin 7, stimulates DNA synthesis in several cell types. Aberrant expression of KRT7 in budding cancer cells represents a modification of the epithelial phenotype (epithelial–epithelial transition) which may be linked to gains in motility and invasive potential (18). KRT7 expression is associated with a higher morbidity and a higher progression in colorectal cancer (18). KRT7 is overexpressed 11.67x more in EAC tumors compared to normal esophageal tissue according to our findings (*P* < 0.0001).”

**LGALS3BP**

“LGALS3BP, or Galectin-3-binding protein, promotes integrin-mediated cell adhesion associated with cancer. This protein has a high affinity for beta-galactoside and has been found to be expressed in many tumor cells associated with aggressive carcinogenesis (19). Breast and lung cancer cells overexpressing LGALS3BP demonstrated resistance to apoptosis in response to cisplatin (20). We found that LGALS3BP is overexpressed at a 3.50x greater level in EAC tumor cells compared to normal esophageal cells (*P* < 0.0001).”

**LTF**

“LTF, or lactotransferrin, stimulates the TLR4-signaling pathway, leading to NFkβ activation and subsequent pro-inflammatory cytokine production while also interfering with the lipopolysaccharide-stimulated TLR4 signaling and also stimulates VEGF-mediated endothelial cell migration and proliferation (21). Malignant transformation of endometrial tissue is associated with overexpressed LTF (22). LTF is expressed at a 9.56x greater level in EAC tumors compared to normal esophageal tissue according to our findings (*P* < 0.0001).”

**PRMT1**

“PRMT1 is the main enzyme that mediates the methylation of histone H4, a specific tag for epigenetic transcriptional activation. PRMT1 has also been identified as a key regulator of the epithelial–mesenchymal transition in breast cancer (36). PRMT1 expression is associated with poor prognosis in gastric cancer patients and has been observed to be significantly upregulated in non-small cell lung carcinoma (23, 24). Knockdown of PRMT1 in three NSCLC cell lines was associated with a significant suppression of cell growth (25). We found PRMT1 to be expressed at a 2.02x greater level in EAC tumor cells compared to normal esophageal epithelial cells (*P* < 0.0001).”

**S100P**

“S100P proteins are localized in the cytoplasm of a wide range of cells and involved in the regulation of several cellular processes such as cell cycle progression and differentiation (34). Significant correlation was found between high expression and S100P and shorter overall survival (OS) and increased drug resistance in gastric and ovarian cancer (28). S100P also plays a key role in the aggressiveness of pancreatic cancer which is likely mediated by its ability to activate RAGE (29). We found S100P to be expressed at a 7.22x greater level in EAC tumors compared to normal esophagus tissue (*P* < 0.0001).”

Corrections have also been made to the **Results**, subsection **Silenced “Good Guys” (Prognostic)**:

**ANXA1**

“ANXA1, or annexin A1, has anti-inflammatory activity and contributes to the adaptive immune response by enhancing signaling cascades that are triggered by T-cell activation (11) (for expression patterns of all six proteins, see Figure 3). Downregulation of ANXA1 is associated with more rapid cancer recurrence in bladder cancer (6 months vs 12 years) (11). Knockdown of ANXA1 was found to block the intake of chemotherapy, leading to anticancer drug resistance (11). Downregulation of ANXA1 has also been associated with radiotherapy resistance and increased relapse rates in head and neck cancer (12). ANXA1 is expressed −8.75x less in EAC tumors compared to normal esophageal tissue exhibiting massive downregulation (*P* < 0.0001).”

**CRNN**

“CRNN, or cornulin, is a survival factor that participates in the proliferation of squamous esophageal epithelial cells and attenuates apoptotic cell death. Heat shock proteins like CRNN play a critical role in controlling unusual environmental pressures placed on squamous epithelial cells (37). Loss of CRNN expression has been correlated with an advanced tumor length, a greater tumor invasion depth, lymph node metastasis, and poor survival in patients with esophageal squamous cell carcinoma (13). On the other hand, patients with high CRNN gene expression were more likely to achieve a pathologic complete response to neoadjuvant chemoradiotherapy (13). However, CRNN is expressed −34.28x less in EAC tumor cells compared to normal esophageal epithelial cells according to our findings (*P* < 0.0001), adding CRNN as a viable oncoprotein for EAC as well.”

**IL-1RA**

“IL-1RA is an endogenous protein that inhibits the activity of interleukin-1 by binding to receptor IL-1R and preventing its association with its co-receptor for signaling (17). Multiple studies indicate that IL-1 plays a role in tumor development and progression, and a high expression of IL-1RA is associated with antitumor activity in various cancer models, including melanoma (17). Also, patients receiving IL-1RA prior to chemotherapy were found to have enhanced treatment responses (17). We found IL-1RA to be expressed −15.34x less in EAC tumors compared to normal esophageal tissue (*P* < 0.0001).”

**S100A8**

“S100A8 has extracellular functions involved in pro-inflammatory, antimicrobial, and apoptosis-inducing activities (38). Calprotectin, a heterodimeric complex of the calcium-binding proteins S100A8 and S100A9, regulates cell cycle progression at G2/M, inhibiting cancer cell migration and invasion, and suppressing tumorigenesis *in vitro* and *in vivo* (26). Downregulation of S100A8 in head and neck squamous cell carcinoma is associated with poor prognosis and lower rates of survival (26). And on the other hand, a high S100A8 expression was found to be a favorable prognostic factor for the survival of oropharyngeal squamous cell carcinoma (38). We found S100A8 to be expressed −7.75x less in EAC tumor cells compared to normal epithelial cells of the esophagus (*P* < 0.0001).”

**SFN**

“SFN, or 14-3-3 sigma, is an adapter protein involved in regulating both general and specialized signaling pathways. Downregulation of SFN has been associated with multistage carcinogenesis and poor prognosis in salivary gland adenoid cystic carcinoma and esophageal squamous cell carcinoma (30, 31). We found SFN to be expressed −10.49x less in EAC tumor tissue compared to normal esophagus (*P* < 0.0001).”

**TXN**

“TXN, or thioredoxin, participates in various redox reactions and catalyzes dithiol–disulfide exchange reactions. Downregulation of TXN in lung cancer results in an increased reactive oxygen species and alters tumor metabolism, resulting in cisplatin resistance (33). We found TXN to be expressed −2.07x less in EAC tumors compared to normal esophageal tissue (*P* < 0.0001).”

Lastly, the T-scores (column 3) in Table 1 were also corrected to display the magnitude of the expression difference. The corrected Table 1 appears below:

**Table 1:** Summary of results: details of the 12 novel markers found to be involved in the pathogenesis of EAC.

**Novel marker**	**Role in EAC progression**	**Expression difference tumor vs normal**	**Tumor vs Normal expression (*P*-value)**	**Specimen with similar expression patterns (Figure 4)**	**Described role as an oncoprotein in other cancers**	**Drug target type**	**Chemoresistance**
ANXA1 (Annexin A1)	Decreases Tumor Suppression	−8.75	1.67E-15	20 out of 20	Downregulation associated with more rapid cancer recurrence (6 months vs 2 years) in bladder cancer (11)	Potential agonist	Downregulation associated with resistance to chemoradiation (11, 12)
CRNN (Cornulin)	Decreases Tumor Suppression	−34.28	0.00	20 out of 20	Downregulation associated with greater tumor length, tumor invasion and lymph node metastasis and lower survival in ESCC (13)	Potential agonist	No evidence found in the literature
HMGB1 (Amphoterin)	Increases Cellular Proliferation	2.33	1.23E-05	19 out of 20	Overexpression associated with poorer prognosis in CRC patients. Inhibition prolonged survival in mesothelioma mice model (14, 15)	Potential antagonist	Overexpression associated with resistance to cisplatin in lung cancer (16)
IL-1RA	Decreases Tumor Suppression	−15.34	2.22E-16	20 out of 20	IL-1RA disrupts IL-1 from playing a role in tumor development and progression and demonstrated antitumor activity in melanoma (17)	Already an antagonist	Downregulation associated with resistance to cisplatin and docetaxel (17)
KRT7 (Keratin 7)	Increases Cellular Proliferation	11.67	5.56E-08	20 out of 20	Overexpression associated with higher morbidity and higher progression in CRC (18)	Potential antagonist	No evidence found in the literature
LGALS3BP (Galectin-3)	Increases Cellular Proliferation	3.50	3.51E-05	20 out of 20	Breast and lung cancer cells overexpressing LGALS3BP show apoptosis resistance in response to cisplatin (19, 20)	Potential antagonist	Overexpression associated with resistance to cisplatin (20)
LTF (Lactoferen)	Increases Cellular Proliferation	9.56	2.65E-06	18 out of 20	Overexpression associated with migration and proliferation in nasopharyngeal carcinoma and endometrial cancer (21, 22)	Potential antagonist	No evidence found in the literature
PRMT1	Increases Cellular Proliferation	2.02	6.50E-05	19 out of 20	Nuclear expression is associated with poor prognosis and chemoresistance in gastric cancer. Upregulated in NSCLC (23–25)	Potential antagonist	Overexpression associated with resistance to cisplatin and 5-FU (23)
S100A8	Decreases Tumor Suppression	−7.75	1.98E-09	20 out of 20	Downregulation is associated with poor prognosis and low rates of survival in head and neck squamous cell carcinoma (26)	Potential agonist	Downregulation associated with resistance to cisplatin and paclitaxel (27)
S100P	Increases Cellular Proliferation	7.22	6.05E-08	19 out of 20	Expression increases is associated w/ poor prognosis and shorter survival in gastric, pancreatic and ovarian cancer (28, 29)	Potential antagonist	Overexpression associated with resistance to 5-fluorouracil (28)
SFN (14-3-3 Sigma)	Decreases Tumor Suppression	−10.49	1.01E-13	20 out of 20	Downregulation correlates with multistage carcinogenesis and poor prognosis in ESCC/ salivary gland adenoid cystic cancer (30, 31)	Potential agonist	Downregulation associated with resistance to cisplatin (32)
TXN (Thioredoxin)	Decreases Tumor Suppression	−2.07	5.96E-06	19 out of 20	Downregulation in lung cancer results in increased ROS and alteration in tumor metabolism resulting in cisplatin resistance (33)	Potential antagonist	Downregulation associated with resistance to cisplatin (33)

*The table includes the expression difference values between normal and tumor cells, the percentage of patients exhibiting these expression patterns, the descriptions of these markers involvement in other cancerous indications, potential drug types to target these markers, and reported associations to chemoresistance (12, 15, 16, 21, 2527, 32, 34). 5-FU, fluorouracil; CRC, colorectal cancer; ESCC, esophageal squamous cell carcinoma; NSCLC, non-small cell lung carcinoma*.

The authors apologize for these errors and state that this does not change the scientific conclusions of the article in any way. The original article has been updated.

